# Compliance with Weekly Iron and Folic Acid Supplementation and Its Associated Factors among Adolescent Girls in Tamale Metropolis of Ghana

**DOI:** 10.1155/2019/8242896

**Published:** 2019-12-11

**Authors:** S. Dajaan Dubik, Kingsley E. Amegah, Amshawu Alhassan, Louis N. Mornah, Loveland Fiagbe

**Affiliations:** ^1^Department of Nutritional Sciences, University for Development Studies, Box TL 1350, Tamale, Northern Region, Ghana; ^2^Department of Health Information, Hohoe Municipal Hospital, Hohoe, Volta Region, Ghana; ^3^Savelugu/Nanton Municipal Health Directorate, Ghana Health Service, Savelugu, Northern Region, Ghana; ^4^Kete-Krachi District Health Directorate, Ghana Health Service, Kete-Krachi, Volta Region, Ghana

## Abstract

**Background:**

In Ghana, anaemia is a severe public health problem among adolescent girls. In an attempt to deal with this phenomenon, Ghana Ministry of Health in collaboration with other development partners developed and launched weekly iron and folic acid supplementation program for adolescent girls in Ghanaian junior high schools. Therefore, the main aim of this study was to determine the level of compliance with iron and folic acid supplementation (IFAS) and its associated factors among adolescent girls in the Tamale Metropolis of Ghana.

**Methods:**

A cross-sectional study was conducted among 424 randomly sampled adolescent girls in the Tamale Metropolis of Ghana from April to July 2019 using an interviewer-administered structured questionnaire. Twenty school health coordinators were purposively selected to answer questions on the challenges they face in implementing the IFAS program at the school level. Bivariate logistic regression and multivariate logistic regression were used to determine associations and strength of associations, respectively, at a significant threshold of *p* < 0.05.

**Results:**

Compliance with the IFAS was low (26.2%). Adolescent girls who were aware of anaemia (AOR = 3.57 (95% CI: 1.96, 6.51) *p* < 0.01), had good knowledge of anaemia (AOR = 1.82 (95% CI: 1.17, 2.81) *p*=0.01), and had good knowledge of the IFAS program (AOR = 2.29 (95% CI: 1.47, 3.57) *p* < 0.01) were significantly associated with compliance with the IFAS. The majority (60%) of the adolescent girls have ever missed taking the iron and folic acid (IFA) tablet because it was not issued to them by the teacher's concern while about 48.3% (169) of the adolescent girls are taking the tablet because it prevents anaemia. Adolescent girls perceiving the tablet as family planning medicine (88.8%) and unavailability of water in classrooms (18.8%) were cited as the major challenges by school health coordinators.

**Conclusion:**

Compliance with the IFAS among adolescent girls was low. Level of education and occupation of mothers of adolescent girls, awareness on anaemia, and good knowledge of anaemia and of the IFAS program were significant predictors of compliance with the IFAS. Educating the adolescent girls on anaemia and benefits of the IFAS, constant supply of the IFA tablet, and engaging parents of the adolescent girls on the program will help improve the compliance level of the adolescent girls with the IFAS.

## 1. Introduction

In developing countries, anaemia is a major public health problem of not only pregnant women or children but also adolescent girls [1]. Adolescence, a period of transition to adulthood, is characterized by intense growth resulting in behavioural and sexual maturity in an individual [2]. It is the second growth spurt of life where girls undergo different experiences. During adolescence, there is an increased demand for nutrition especially iron requirements [2]. Adolescence is also considered as a golden time for interventions to curb anaemia. It also presents the right time for building a nutrition foundation for child bearing in later life [3].

Globally, anaemia is affecting about 1.62 billion people worldwide and approximately half of all anaemia can be attributed to iron deficiency. Children, pregnant women, and women of reproductive age are greatly affected [4]. The number of nonpregnant women of reproductive age affected by anaemia increased from 464 million in 2000 to 578 million in 2016 across the globe. Africa and Asia are the hardest hit with the prevalence of over 35% and therefore require increased efforts to curb the problem [5].

Worldwide, it is estimated that the absence of investment in prevention of anaemia would result in 265 million more cases of anaemia in women including adolescent girls by 2025 and nearly 800,000 more child deaths and 7,000–14,000 more maternal deaths [6].

Anaemia, a direct indication of undernutrition and poor dietary intake of iron, is a serious public health problem among adolescent girls [2]. Adolescent girls are at greater risk of iron deficiency and anaemia due to increased growth, poor dietary intake of iron, low bioavailability of dietary iron, and high rate of infectious diseases, parasitic infections, and menstrual blood loss [7]. Iron deficiency anaemia occurs more often in adolescent girls than in adolescent boys. This is due to excessive loss of iron during menstruation. Moreover, the risk of anaemia in adolescent girls is increased by poor literacy, ignorance, and lack of knowledge about iron deficiency [8].

Anaemia has serious consequences; it can lead to poor school performance among schoolchildren, impaired physical growth, and poor cognitive development, thereby hindering development socially and economically [4]. Anaemia can also leave adolescent girls physically inactive, hindering their progress in school and ultimately causing them to drop out of school [3]. The ultimate aim of managing anaemia is to help elevate the haemoglobin level of the individual concern, and this is mostly done through iron therapy and blood transfusion [9]. However, at the population level, iron deficiency anaemia can be tackled through deworming, iron supplementation, fortification of foods with micronutrients, and proper dietary education with improved food security [3, 10].

In Ghana, the prevalence of anaemia among adolescent girls is unacceptably high. Data from the Ghana Demographic Health Survey (GDHS) indicate a high prevalence of 48% among adolescent girls [11]. Iron and folic acid supplementation are known as the most cost-effective way of improving the iron status of adolescent girls in developing countries [8]. Therefore, constant use of iron and folic acid supplements together with a diet rich in micronutrients is essential for the prevention of iron deficiency anaemia among adolescent girls [2]. In addition, the effectiveness of weekly supplementation of IFA in preventing anaemia among adolescent girls has been documented in several studies [12–15]. Taking cognizance of this, Ghana Ministry of Health in 2017 with support from the Ministry of Education and other development partners has launched a weekly IFAS program in order to meet the challenge of the high prevalence of anaemia among adolescent girls in Ghana. However, the major problem with the IFAS program is compliance with several interacting factors that still remain unclear in our study area [16]. Furthermore, the IFAS program is relatively new among adolescent girls in Ghana. Therefore, the aim of this study was to determine the level of compliance with the IFAS and its influencing factors among adolescent girls in the Tamale Metropolis of Ghana. The specific aims of the present study were to (1) determine compliance level of the adolescent girls with the IFAS, (2) assess factors influencing compliance with the IFAS, (3) determine the adolescent girls' level of knowledge on anaemia and the IFAS, and (4) assess challenges faced by teachers in implementing the program at the school level.

## 2. Methods

### 2.1. Study Area and Design

A cross-sectional study was conducted using quantitative data collection methods from April to July 2019 among adolescent girls in twenty selected junior high schools in the Tamale Metropolis of Ghana. Tamale Metropolis is geographically located between latitude 9° 16 and 9° 34 North and longitudes 0° 36 and 0° 57 West. Tamale Metropolis is one of the 14 Districts in the Northern Region. It is located in the central part of the region and shares boundaries with Sagnarigu, Mion, East Gonja, and Central Gonja districts. The Metropolis has a total estimated land size of 646.90180 km^2^ [17]. According to the Ghana 2010 population and housing census, Tamale Metropolis has a population of 233,252 constituting 49.7% males and 50.3% females. Among those aged 3 years and older and are currently attending school, 18.2% are in junior high schools. The metropolis has 112 junior high schools [17].

### 2.2. The Weekly IFAS Program among Adolescent Girls in Ghana

In Ghana, IFAS has been for only pregnant women neglecting adolescent girls [3]. Weekly IFAS program which is the first of its kind in Ghana among adolescent girls was launched in 2017 by Ghana Ministry of Health with support from development partners such as UNICEF and WHO. The initiative is expected to reach about 360,000 girls in junior and senior high schools and 600,000 girls who are out of school [18].

The IFAS program is in the pilot phase in four of the ten regions, namely, Northern Region, Brong Ahafo Region, and Upper West and Upper East regions with the main aim of contributing to the reduction of anaemia among adolescent girls. About 4,500 school health coordinators who are teachers in the participating schools and 3,000 health workers have been trained to support the implementation of the program [18]. Teachers are expected to encourage adolescent girls in school to take one supplement (60 mg of elemental iron and 400 *μ*g (0.4 mg) of folic acid) every Wednesday in a week at noon. Community health workers are expected to assist girls who are out of school to take the tablet [3].

### 2.3. Study Population

All Adolescent girls attending junior high school in Tamale Metropolis were the source population. The study population consisted of randomly sampled girls who were attending junior high school in the study area in the third term of the 2018/2019 academic year. Girls who were absent and those who declined to answer the research questions were excluded from the study. School health coordinators of the 20 schools also participated in the study.

### 2.4. Sample Size Determination and Sampling Procedures

The sample size was determined using Cochran's formula *N*=(*Z*2  × *p*(1 − *p*))/*d*2 [19] with 95% confidence interval and 5% margin error with assumed compliance rate of 50%, where *N* is the sample size, *Z* (statistic) = 1.96, *p* (compliance rate) = 0.5, and d (margin of error) = 0.05:

(1) $$\begin{array}{c} n=\frac{\left(1.96\right)2\;\times 0.5\left(1-0.5\right)}{\left(0.05\right)2}=384.16. \end{array}$$

The IFAS program is relatively a new program among adolescent girls in Ghana, hence the justification for the assumed compliance rate for the sample size calculation. The calculated sample size was therefore 385. After adding a 10% nonresponse rate, the final sample size obtained was 424 research participants.

Simple random sampling was used to select the schools and the adolescent girls while purposive sampling was used to select the school health coordinators. Twenty schools were selected using lottery method. The names of all junior high schools in the metropolis were written on pieces of papers and placed in a bowl. With blind draw, the schools were randomly picked one by one without replacement until 20 schools were reached. The final sample size of 424 was proportionally allocated to the 20 schools based on the enrolment of girls in each school. The study participants were selected from each class through simple random sampling using random numbers generated for each class using the girls attendance register. Twenty school health coordinators in the selected schools were purposively sampled to participate in the study.

### 2.5. Data Collection

The data collection tool used in collecting the research data was structured pretested questionnaires while data collection was done with the help of interviewer-assisted questionnaire administration. Pretesting of the data collection tool was done in Sagnerigu Municipality since it shares similar characteristics with the study area. The data collection tool was adapted and modified from similar published studies [1, 2, 7, 20]. The adoption of the data collection tool from similar published studies was to ensure the validity of the data collection tool. Thirty adolescent girls were conveniently chosen in one school for the pretesting of the data collection tool. Pretesting was done to ensure the reliability of the data collection tool. Questions that gave responses that were not certain were reframed and tested again on the same respondents to ensure their reliability.

A prior appointment was taken from headmasters and health coordinators of the selected schools. The questionnaires which were mostly closed-ended with multiple choice and dichotomous responses were divided into sections and administered to both adolescent girls and teachers. Section A consisted of 8 questions to elicit information on sociodemographic characteristics of the adolescent girls and section B was made of 9 questions to determine compliance level of the adolescent girls with the IFAS whereas section C consisted of 16 questions which sought to assess the level of knowledge of the adolescent girls of anaemia and of the IFAS program. Section C also sought to find out the side effects experienced by adolescent girls. Section D which was the only section administered to the teachers sought to determine the challenges faced by teachers in implementing the IFAS program at the school level. Data collection was done by two community health nurses and two masters students in public health nutrition. One-day training was organized for the data collectors by the principal investigator. They were trained on objectives of the study, how to ensure confidentiality of the data, and how to fill the questionnaires. They were also trained on how to initiate the interview process by reading the informed consent to every respondent for voluntary participation. After data collection, the principal investigator reviewed each questionnaire in order to ensure the accuracy and completeness of the data collected.

### 2.6. Statistical Analysis

The quantitative data were coded and analysed using STATA 14.1. Sociodemographic characteristics, level of compliance, and knowledge of anaemia and of the IFAS program were first presented in text, figures, and tables using descriptive statistics such as frequencies and percentages. Bivariate logistic regression test was done to determine the association between the sociodemographic characteristics and the outcome variables (compliance level, knowledge of anaemia, and knowledge of the IFAS program). Logistic regression analysis was also done to determine the effect of awareness of anaemia, knowledge of anaemia, and knowledge of the IFAS program on compliance level. Multivariate logistic regression was further done for variables that were statistically significant after the bivariate analysis. Crude and adjusted odds ratios with their respective confidence intervals (95%) were computed to determine the strength of association of each variable.

IFAS compliance level was measured based on the number of tablets consumed in the past 7 weeks. Adolescent girls who consumed at least five tablets of the expected dose in the previous 7 weeks (1 tablet per week) which is equivalent to consuming 70% of the expected dose before the day of the data collection was considered compliant. Adolescent girls who consumed less than five tablets were considered noncompliant [16]. Records kept by the school health coordinators were used to validate each respondent's compliance status.

Knowledge on anaemia was measured by summing up 19 relevant knowledge items on anaemia (1 item on the meaning of anaemia, 4 items on causes of anaemia, 6 items on signs and symptoms of anaemia, 4 items on consequences of anaemia, and 4 items on prevention of anaemia). A correct response was scored “1” and an incorrect response was scored “0” with a maximum score of 19. Those who scored above the median mark were considered as having good knowledge of anaemia while those who scored below the median mark were considered as having poor knowledge [16].

Knowledge of the IFAS program was scored by summing up 15 relevant knowledge items on the IFAS program: one item on why they are given the tablet, 4 items on benefits of taking IFA tablet, 3 items on food that inhibits iron and folic acid absorption, 5 items on side effects of taking IFA tablets, and 2 items on why girls are given the iron and folic acid tablet. A correct answer was scored “1” and an incorrect answer was scored “0”. With a maximum score of 15, those who scored above the median mark were considered having good knowledge on the IFAS program while those who scored below the median mark were considered as having poor knowledge [21].

### 2.7. Ethical Consideration

Ethical clearance for the study was sought from the University for Development Studies. Approval letter for the study was granted by Ghana Health Service while permission to go into the school was obtained from the Tamale Metropolitan Education Office. Informed assent was obtained from underaged participants in their schools while informed consent was obtained from the parents of the girls. Selected girls were given informed consent forms for their parents to thumbprint/sign signalling their agreement for their child to participate in the study.

## 3. Results

### 3.1. Sociodemographic Characteristics of the Adolescent Girls

The total number of respondents in this current study was 424 with a mean age (SD) of 14.4 (±1.7). Majority (79.7%) of the respondents had their ages ranging between 10 and 15 years with over half (53.5%) in junior high school two. Majority (67.9%) of the adolescent girls were Dagombas with 73.6% of them affiliated to the Islamic religion. Most (48.1%) of the respondents' mothers had no formal education while very few (14.2%) had tertiary education, and about half (51.9%) of the respondents' mothers were petty traders. Twenty-five percent of the respondents' fathers had tertiary education while most (44.8%) of them had no formal education with 46% of them being public/civil servants (Table 1).

### 3.2. Compliance with the IFAS

Overall compliance with the IFAS in this current study was 26.2%. Only 26.2% (111) of the adolescent girls consumed 5 or more tablets in the past 7 weeks prior to the data collection. Majority (90.1%) of the adolescent girls took the IFA tablet on the first day it was given by the teachers. About 82.5% of the adolescent girls are still consuming the IFA tablet in school while the rest (17.4%) stopped consuming the IFA tablet. Among those currently taking the IFA tablet in school, most (48.3%) cited the prevention of anaemia as the reason for taking the IFA tablet while another 41.1% cited advice from their teacher. Among those who stopped taking the IFA tablet in school, 90.5% (67) cited the fact that their parents ask them not to take the tablet while another 5.4% (4) cited side effects as the reason for ceasing to take the IFA tablet. About 82.5% of the adolescent girls have ever missed taking the IFA tablet in school. Most (60%) of the adolescent girls have ever missed taking the IFA tablet because it was not issued to them by the teacher's concern while another 28.9% cited the fact that they were absent from school. Among those who have ever taken the tablet, 73.1% always take the tablet in the presence of the teacher (Table 2).

A Pearson's chi-square test with a significant threshold of *p* < 0.05 established that the mother's level of education (*χ*^2^ = 15.65, *p* < 0.01) and mother's occupation (*χ*^2^ = 10.30, *p*=0.02) were significantly associated with adolescent girls level of compliance (Table 3). Respondents whose mothers had secondary education were 2.1 times more likely to comply with the IFAS as compared to those respondents whose mothers had no formal education (OR = 2.14 (95% CI: 1.17, 3.29) *p*=0.01). The odds of compliance with the IFAS among respondents whose mothers were traders were 56% times lower compared to those respondents whose mothers were unemployed (AOR = 0.44 (95% CI: 0.23, 0.85) *p*=0.01) (Table 4). Good knowledge of anaemia (OR = 1.82 (95% CI: 1.17, 2.81) *p*=0.01) and good knowledge of the IFAS program (OR = 2.29 (95% CI: 1.47, 3.57) *p* < 0.01) were also significant determinants of compliance with the IFAS. A logistic regression analysis found out that the odds of compliance with the IFAS were 3.6 times more likely among adolescent girls who were aware of anaemia compared to adolescent girls who were not aware of anaemia (AOR = 3.57 (95% CI: 1.96, 6.51) *p* < 0.01) (Table 5).

### 3.3. Perceived Benefits of Taking the IFA Tablet as Reported by Adolescent Girls

**Figure 1:**
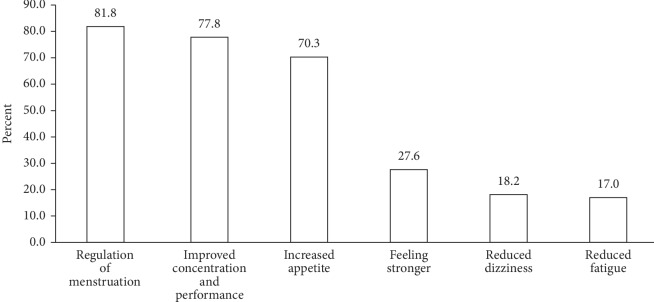
Perceived benefits of consuming IFA tablets.

With multiple responses possible, majority (81.8%) of the adolescent girls cited regulation of menstruation as the benefit they have gotten from taking the IFA tablet and 77.8% thinks the IFA tablet has helped to improve their concentration and performance in class while over 18.2% cited reduced dizziness as the benefit they have gotten from taking the IFA tablet (Figure 1).

### 3.4. Awareness of Anaemia among Adolescent Girls

About 247 (58.2%) of the respondents claimed they have ever heard of a disease where the affected person is said to have low blood level, and only 30.7% (130) were able to correctly mention anaemia as low blood level and hence were considered aware of anaemia. About 67.9% of the adolescent girls have never received education on anaemia. Those (32.1%) who have ever received education on anaemia cited teachers (85.3%) and health workers (14.7%) as their sources of anaemia education.

### 3.5. Factors Associated with Adolescent Girls' Level of Knowledge on Anaemia

**Figure 2:**
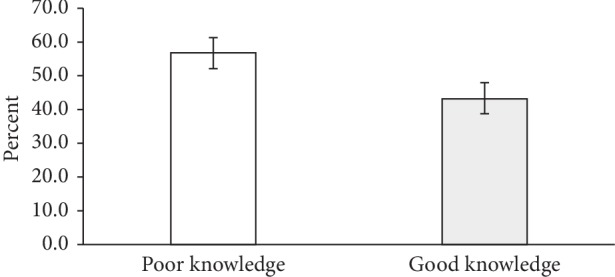
Respondents' knowledge of anaemia.

In this current study, it was shown that more than half (56.8%) of the adolescent girls had poor knowledge on anaemia while 43.2% had good knowledge of anaemia (Figure 2). Pearson chi-square test with a significance threshold of *p* < 0.05 found no statistically significant association between respondent sociodemographic characteristics and knowledge on anaemia except for the occupation of fathers of the respondents (*χ*^2^ = 11.46, *p* < 0.01). Respondents whose fathers were public/civil servants were found to be 4.4 times more likely to have good knowledge on anaemia as compared to those pupils whose fathers were unemployed (OR = 4.4 (95% CI: 1.4, 13.5) *p*=0.01) (Table 6).

### 3.6. Adolescent Girls' Level of Knowledge on the IFAS Program

**Figure 3:**
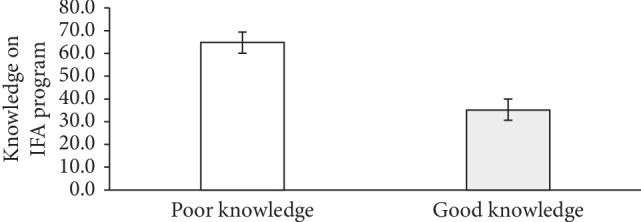
Respondents' knowledge of the IFAS program.

Most (64.9%) of the adolescent girls were found to have poor knowledge of the IFAS program while 35.1% were found to have good knowledge of the IFAS program (Figure 3). Pearson chi-square test with a significance threshold of *p* < 0.05 found out that mother's level of education (*χ*^2^ = 9.92, *p* < 0.02), mother's occupation (*χ*^2^ = 8.39, *p* < 0.04), and father's occupation (*χ*^2^ = 10.88, *p* < 0.01) of the respondents were statistically significant with adolescent girls' level of knowledge on the IFAS program (Table 7). Respondents whose mothers had secondary education were 2.1 times more likely to have good knowledge of the IFAS as compared with those whose mothers had no formal education (OR = 2.1 (95% CI: 1.16, 3.77) *p*=0.01). Again, respondents whose fathers were unemployed were 75% times less likely to have good knowledge of the IFAS program as compared to those respondents whose fathers were public/civil servants (OR = 0.25 (95% CI: 0.07, 0.88) *p*=0.03).

After adjusting for the effect of all confounding variables in a logistic regression model, the mother's education remained statistically significant. Respondents whose mothers had secondary education were 2.2 times more likely to have good knowledge of the IFAS program as compared to those respondents whose mothers had no formal education (OR = 2.19 (95% CI: 1.81, 4.07) *p*=0.01) (Table 8).

### 3.7. Side Effects Reported by the Adolescent Girls

**Figure 4:**
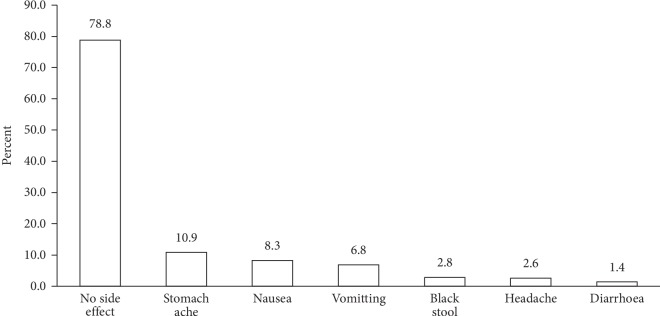
Respondents' perceived side effects of IFA supplement.

With multiple responses possible, most (78.8%) of the respondents reported that there were no side effects associated with the consumption of the IFA tablet. Some (10.9%) stated stomach/abdominal pains as the side effect they experienced with the consumption of the IFA tablet while few cited nausea (8.3%) (Figure 4).

### 3.8. Challenges Faced by Teachers in Implementing the IFA Supplementation Program

At the time of our visit, about 70% of the schools had IFA tablets in stock with school health coordinators (95%) in charge of issuing the IFA tablet to the adolescent girls. About 45% (9) of the school health coordinators have never educated the adolescent girls on anaemia and on the IFAS program. Among those who have never educated the adolescent girls on anaemia, 77.8% (7) cited limited time as one of the reasons for their inability to educate the adolescent girls on anaemia and on the IFAS program.

A significant number (70%, 14) of the school health coordinators were not able to organize training for their colleague teachers with half (50%, 7) of them citing the fact that they can handle the work alone while another half reported there was no time to organize the training for colleague teachers. Fifty-five percent of the school health coordinators do not always supervise the adolescent girls to ingest the tablet with the majority 63.6% of them citing limited time for their inability to supervise them. Unavailability of water in the classroom (36.4%) was also cited as one of the reasons for their inability to always supervise the adolescent girls to ingest the tablet. About 80% of the school health coordinators admit the fact that they face challenges in implementing the IFAS program. Adolescent girls refusing to take the IFA tablet because they perceived it as family planning medicine and lack of water to swallow the tablet were some of the challenges opined by the school health coordinators. School health coordinators think educating parents and children on the IFAS program, providing incentives to school health coordinators, and ensuring the availability of water in classrooms could help solve some of these challenges.

## 4. Discussion

In this study, we sought to investigate the compliance level of adolescent girls with the IFAS and its influencing factors among adolescent girls in the Tamale Metropolis of Ghana. We included 424 adolescents girls with a mean age (SD) of 14.4 (±1.7) with the majority of the adolescent girls between 10 and 15 years.

Compliance with the IFAS in this study was found to be low (26.2%). This finding is in variance with a similar study conducted in Iran [22] which found compliance rate to be 62.3% and another study conducted in rural India which also found a compliance rate of 85.8% [20]. However, our study finding is similar to that of Sajna and Jacob [23] which also found compliance to be low (15%). Low compliance in this study could be a result of stock-outs of the IFA tablet as some of the schools had no IFA tablet in stock at the time of our visit. In addition, compliance with the IFAS was affected by absenteeism on the part of the adolescent girls and teachers not issuing the IFA tablet to the adolescent girls. Hence, adolescent girls who are regular in school can be used to always remind the teachers in charge of the IFAS program to issue the IFA tablet to them every Wednesday.

Prevention of anaemia and advice from teachers were found to be the motivating factors for consuming the IFA tablet by the adolescent girls. These findings agree with a cross-sectional study conducted by Dhikale et al. [20] which also found teachers as the motivating factor for the consumption of IFA tablets by schoolchildren. Therefore, educating adolescent girls and teachers on the IFAS program including its benefits will help facilitate regular consumption and compliance with the IFAS by the adolescent girls. It is important to note that compliance with the IFA regimen is very crucial for every IFAS program to achieve its intended objectives [24]. Perhaps, low compliance level in this current study might be a barrier for the program to achieve its objective of addressing anaemia among adolescent girls in the study area.

In this study, level of education and occupation of mothers of adolescent girls, awareness of anaemia, and good knowledge of anaemia and of the IFAS program were statistically associated with compliance with the IFAS. A similar study in Ethiopia also found good knowledge of anaemia and of the IFAS program as predictors of compliance [21]. Lack of knowledge or not receiving counselling on anaemia is associated with noncompliance with IFAS [25]. In this study, the odds of complying with the IFAS were 2.1 times more likely among adolescent girls whose mothers had secondary education compared to adolescent girls whose mothers had no formal education. Therefore, ensuring high compliance in our study area will include the mounting of effective health education strategies in the schools in order to create awareness on anaemia, increase the adolescent girls' level of knowledge of anaemia and of the IFAS program. Again, engaging mothers of adolescent girls will also contribute to the success of the IFAS program.

About 90.5% (67) of the adolescent girls stopped taking the IFA tablet in school because their parents asked them not to take the IFA tablet. This finding agrees with a study conducted in India [1] which also identified parental resistance as one of the reasons for schoolchildren's refusal to take IFA tablet. Perhaps, parents were not engaged before enrolling in the IFAS program in the schools. Engaging parents of the adolescent girls on the IFAS program during Parent Teacher Association (PTA) meetings in the schools is crucial for the success of the program. Less than half of the adolescent girls in this current study report taking the tablet without the supervision of teachers. This deviates from the IFAS program recommendation which requires teachers to directly supervise the adolescent girls to swallow the IFA tablet. Supervising schoolchildren to ingest the IFA tablet is considered one of the most important aspects of the IFAS program in schools. Without supervision, the possibility of schoolchildren throwing the tablet away is very high [26].

Anaemia among adolescent girls can lead to problems such as decreased concentration, poor school performance, weakness, and irregular menstruation. Weekly IFAS is therefore indicated to improve the iron status of adolescent girls and to counteract the negative effects of iron deficiency anaemia [9]. Most of the adolescent girls in this study claimed intake of the IFA tablets has helped to improve their menstruation, concentration, and performance in school. This finding agrees with the studies of Priya et al. [27] and Vishal et al. [15] conducted in an urban setting in India which also found improved menstruation, concentration, and performance in school as positive effects of taking IFA tablet. Girls with these positive experiences could be used as role models to share their own experiences with their colleagues in order to improve on the success of the IFAS program.

We found a poor level of knowledge of the adolescent girls on anaemia. This finding means that adolescent girls' level of knowledge on meaning, causes, consequences, signs and symptoms, and prevention of anaemia altogether was inadequate. This was not surprising since the majority of the adolescent girls have never received education on anaemia. Our findings agree with studies conducted in Ethiopia (43.3%) [28] and Delhi (28.5%) [29] but differ from a study conducted in India (90.5%) [1]. Jalambo et al. in their study in Palestine also concluded that adolescent girls have poor knowledge of anaemia including its causes, prevention, and management [30]. Awareness and having good knowledge of anaemia have the potential to influence adolescent girls' consumption of the IFA tablets [12]. Another study in Ethiopia emphasized the fact that knowledge of anaemia is protective against the risk of anaemia among adolescent girls [28]. Further analysis revealed that the odds of having good knowledge of anaemia among adolescent girls whose fathers were public/civil servants were 4.4 times more likely as compared to those adolescent girls whose fathers were unemployed. This might be due to the fact that public/civil servants are mostly educated and might have discussed anaemia and its related programs with their adolescent girls at home.

We also found a poor level of knowledge of the adolescent girls on the IFAS program. A similar program evaluated in India also found poor knowledge of adolescent girls on IFAS pertaining to side effects of the IFA tablet (29%) and unawareness of benefits (32%) of taking the IFA tablet [26]. Poor level of knowledge on the IFAS program might be due to adolescent girls not receiving education on the IFAS program from their teachers. As part of the implementation of the IFAS program in the schools, school health coordinators were supposed to educate the adolescent girls on anaemia and on the IFAS program before the start of the program.

Majority of the adolescent girl report no side effects associated with the consumption of the IFA tablet. However, popularly reported side effects were stomachache, nausea, and vomiting. Stomach ache, nausea, and vomiting are popularly reported as side effects associated with the consumption of IFA tablets in other studies [7, 22, 26, 31]. Side effects of the IFA tablet are one of the main reasons for noncompliance with IFAS [7, 22]. The success of the IFAS program could be threatened by these side effects.

Unavailability of water in classrooms was seen as an obstacle for teachers to supervise the adolescent girls to ingest the tablet. This finding disagrees with a study conducted in Iran [22] where students had free and unlimited access to water to ingest the IFA tablet but agrees with a systematic review done by Apriani and Syafiq [24] which found access to water as a challenge in implementing IFAS programs in schools. In the absence of water, adolescent girls are given the IFA tablet to go in search of water in order to swallow it.

According to the school health coordinators, the major challenge they face in implementing the IFAS program is the refusal of adolescent girls to take the IFA tablet as the perceived tablet as family planning medicine. This perception may be due to the poor engagement of the adolescent girls on the IFAS program. The fact that the program does not include adolescent boys might also contribute to this misconception about the IFAS. This calls for effective education on the IFAS program in order to enlighten the adolescent girls on its intended benefits and to also debunk the misconceptions the adolescent girls have about the IFA tablet.

## 5. Conclusion

There was low compliance with the IFAS program among adolescent girls. Teachers not issuing the tablet to the adolescent girls and absenteeism on the part of the adolescent girls were seen as the major barriers to compliance. Mother's level of education, mother's occupation, awareness of anaemia, and good knowledge of both anaemia and the IFAS program were found to be significant determinants of compliance. Limited time on the part of the school health coordinators, unavailability of water in classrooms, adolescent girls perceiving the IFA tablet as family planning medicine, and irregular supply of IFA tablet were seen as the major challenges faced by school health coordinators. Educating the adolescent girls on anaemia and the IFAS program, engaging parents of the adolescent girls during PTAs meetings, ensuring unlimited access to water in classrooms and regular supply of the IFA tablet to the schools are recommended in order to ensure the success of the IFAS program in the schools.

## 6. Limitation of the Study

Most of the results in this study were self-reported by the adolescent girls. There is a possible tendency to recall bias and exaggeration. The result should be interpreted with caution. The study did not also measure Hb levels of the adolescent girls to determine the impact of the IFAS on their Hb levels.

## Figures and Tables

**Table 1:** Sociodemographic characteristics of respondents.

Variables	Frequency, *N* = 424	Percent (%)
Mean age (SD)	14.4 (1.7)	

Age group (years)		
10–15	338	79.7
16–20	86	20.3

Class		
JHS 1	197	46.5
JHS 2	227	53.5

Ethnicity		
Dagomba	288	67.9
Mamprusi	24	5.7
Others	112	26.4

Religion		
Christianity	112	26.4
Islam	312	73.6

Mother's level of education		
No formal education	204	48.1
Basic	100	23.6
Secondary	60	14.2
Tertiary	60	14.2

Father's level of education		
No formal education	190	44.8
Basic	50	11.8
Secondary	78	18.4
Tertiary	106	25.0

Mother's occupation		
Unemployed	56	13.2
Public/civil servant	135	31.8
Trader	220	51.9
Retired	13	3.1

Father's occupation		
Unemployed	21	5.0
Public/civil servant	195	46.0
Farmer	185	43.6
Retired	23	5.4

**Table 2:** Compliance with IFAS among adolescent girls.

Variables	Frequency, *N* = 424	Percent (%)
Took IFA tablet on the first day		
No	42	9.9
Yes	382	90.1

Currently taking IFA tablet in school		
No	74	17.5
Yes	350	82.5

Reasons for taking IFA tablet		
Advice from teacher	144	41.1
Because it is free	12	3.4
Friends are taking it	25	7.1
It prevents anaemia	169	48.3

Reasons for not taking the tablet		
Fear of side effects	4	5.4
My parents ask me not to take the tablet	67	90.5
l feel healthy	3	4.1

Number of IFA tablets taken in the past 7 weeks		
<5 tablets	313	73.8
≥5 tablets	111	26.2

Always takes IFA tablet under supervision		
No	114	26.9
Yes	310	73.1

Ever missed taking IFA tablet in school		
No	74	17.5
Yes	350	82.5

Reasons for missing		
Bad taste of tablet	23	6.6
I was absent	101	28.9
Side effects	16	4.6
I was not given IFA tablet	210	60.0

**Table 3:** Sociodemographic characteristics and compliance level among adolescent girls.

Dependent variables	Compliance with IFAS			
	Noncompliance	Compliance	Total, *N* = 424	Chi-square (*p* value)
Age group (years)				
10–15	256 (75.7)	82 (24.3)	338 (100)	3.18 (0.08)
16–19	57 (66.3)	29 (33.7)	86 (100)	

Class				
JHS 1	143 (72.6)	54 (27.4)	197 (100)	0.29 (0.59)
JHS 2	170 (74.9)	57 (25.1)	227 (100)	

Ethnicity				
Dagomba	212 (73.6)	76 (26.4)	288 (100)	0.03 (0.99)
Mamprusi	18 (75)	6 (25)	24 (100)	
Others	83 (74.1)	29 (25.9)	112 (100)	

Religion				
Christianity	77 (68.8)	35 (31.3)	112 (100)	2.03 (0.16)
Islam	236 (75.6)	76 (24.4)	312 (100)	

Mother's level of education				
No formal education	153 (75)	51 (25)	204 (100)	**15.65 (<0.01)**
Basic	85 (85)	15 (15)	100 (100)	
Secondary	35 (58.3)	25 (41.7)	60 (100)	
Tertiary	40 (66.7)	20 (33.3)	60 (100)	

Father's level of education				
No formal education	145 (76.3)	45 (23.7)	190 (100)	6.03 (0.11)
Basic	42 (84)	8 (16)	50 (100)	
Secondary	53 (67.9)	25 (32.1)	78 (100)	
Tertiary	73 (68.9)	33 (31.1)	106 (100)	

Mother's occupation				
Unemployed	35 (62.5)	21 (37.5)	56 (100)	**10.30 (0.02)**
Public/civil servant	94 (69.6)	41 (30.4)	135 (100)	
Trader	176 (80)	44 (20)	220 (100)	
Retired	8 (61.5)	5 (38.5)	13 (100)	

Father's occupation				
Unemployed	18 (85.7)	3 (14.3)	21 (100)	7.81 (0.05)
Public/civil servant	132 (67.7)	63 (32.3)	195 (100)	
Farmer	146 (78.9)	39 (21.1)	185 (100)	
Retired	17 (73.9)	6 (26.1)	23 (100)	

**Table 4:** Association between the odds of sociodemographic characteristics and compliance level among adolescent girls.

Independent variables	Frequency (%)	Unadjusted			Adjusted		
		OR	95% CI	*p* value	OR	95% CI	*p* value
Mother's education							
No formal education	204 (48.1)	Reference					
Basic	100 (23.6)	0.53	0.28, 1.00	0.05	0.51	0.27, 0.96	**0.04**
Secondary	60 (14.2)	2.14	1.17, 3.29	**0.01**	1.96	1.06, 3.63	**0.03**
Tertiary	60 (14.2)	1.50	0.80, 2.80	0.20	1.24	0.62, 2.48	0.54

Mother's occupation							
Unemployed	56 (13.2)	Reference					
Public/civil servant	135 (31.8)	0.73	0.34, 0.38	0.34	0.69	0.34, 1.42	0.32
Trader	220 (51.9)	0.42	0.22, 0.79	**0.01**	0.44	0.23, 0.85	**0.01**
Retired	13 (3.1)	1.04	0.30, 3.60	0.95	1.08	0.30, 3.92	0.90

**Table 5:** Factors associated with IFAS compliance level among adolescent girls.

Independent variables	Frequency (%)	Unadjusted			Adjusted		
		OR	95% CI	*p* value	OR	95% CI	*p* value
Awareness of anaemia							
No	294 (69.3)	Reference					
Yes	130 (30.7)	4.04	2.25, 7.25	**<0.01**	3.57	1.96, 6.51	**<0.01**

Knowledge of anaemia							
Poor	241 (56.8)	Reference					
Good	183 (43.7)	1.82	1.17, 2.81	**0.01**	1.12	0.63, 2.00	0.70

Knowledge of the IFAS program							
Poor	275 (64.9)	Reference					
Good	149 (35.1)	2.29	1.47, 3.57	**<0.01**	1.67	0.94, 3.00	0.08

**Table 6:** Association between respondents' sociodemographic characteristics and knowledge on anaemia.

Independent variables	Knowledge on anaemia				Unadjusted OR (95% CI) *p* value
	Poor knowledge	Good knowledge	Total, *N* = 424	Chi-square (*p* value)	
Age group (years)					
10–15	192 (56.8)	146 (43.2)	338 (100)	0.00 (0.98)	
16–20	49 (57)	37 (43)	86 (100)		

Class					
JHS 1	114 (57.9)	83 (42.1)	197 (100)	0.16 (0.69)	
JHS 2	127 (55.9)	100 (44.1)	227 (100)		

Ethnicity					
Dagomba	168 (58.3)	120 (41.7)	288 (100)	0.82 (0.67)	
Mamprusi	13 (54.2)	11 (45.8)	24 (100)		
Others	60 (53.6)	52 (46.4)	112 (100)		

Religion					
Christianity	63 (56.3)	49 (43.8)	112 (100)	0.02 (0.88)	
Islam	178 (57.1)	134 (42.9)	312 (100)		

Mother's level of education					
No formal education	124 (60.8)	80 (39.2)	204 (100)	5.16 (0.16)	
Basic	59 (59)	41 (41)	100 (100)		
Secondary	30 (50)	30 (50)	60 (100)		
Tertiary	28 (46.7)	32 (53.3)	60 (100)		

Father's level of education					
No formal education	116 (61.1)	74 (38.9)	190 (100)	5.14 (0.16)	
Basic	31 (62)	19 (38)	50 (100)		
Secondary	37 (47.4)	41 (52.6)	78 (100)		
Tertiary	57 (53.8)	49 (46.2)	106 (100)		

Mother's occupation					
Unemployed	28 (50)	28 (50)	56 (100)	6.41 (0.09)	
Public/civil servant	68 (50.4)	67 (49.6)	135 (100)		
Trader	136 (61.8)	84 (38.2)	220 (100)		
Retired	9 (69.2)	4 (30.8)	13 (100)		

Father's occupation					
Unemployed	17 (81)	4 (19)	21 (100)	**11.46 (0.01)**	*Reference*
Public/civil servant	96 (49.2)	99 (50.8)	195 (100)		4.4 (1.4, 13.5) **0.01**
Farmer	114 (61.6)	71 (38.4)	185 (100)		2.6 (0.9, 8.2) 0.09
Retired	14 (60.9)	9 (39.1)	23 (100)		2.7 (0.7, 10.8) 0.15

**Table 7:** Association between sociodemographic characteristics and knowledge on the IFAS program.

Independent variables	Knowledge on the IFAS program			Chi-square (*p* value)
	Poor knowledge	Good knowledge	Total, *N* = 424	
Age group (years)				
10–15	217 (64.2)	121 (35.8)	338 (100)	0.32 (0.57)
16–20	58 (67.4)	28 (32.6)	86 (100)	

Class				
JHS 1	122 (61.9)	75 (38.1)	197 (100)	1.39 (0.24)
JHS 2	153 (67.4)	74 (32.6)	227 (100)	

Ethnicity				
Dagomba	190 (66)	98 (34)	288 (100)	4.04 (0.13)
Mamprusi	11 (45.8)	13 (54.2)	24 (100)	
Others	74 (66.1)	38 (33.9)	112 (100)	

Religion				
Christianity	76 (67.9)	36 (32.1)	112 (100)	0.60 (0.44)
Islam	199 (63.8)	113 (36.2)	312 (100)	

Mother's level of education				
No formal education	141 (69.1)	63 (30.9)	204 (100)	9.92 **(0.02)**
Basic	70 (70)	30 (30)	100 (100)	
Secondary	31 (51.7)	29 (48.3)	60 (100)	
Tertiary	33 (55)	27 (45)	60 (100)	

Father's level of education				
No formal education	130 (68.4)	60 (31.6)	190 (100)	7.68 (0.05)
Basic	38 (76)	12 (24)	50 (100)	
Secondary	47 (60.3)	31 (39.7)	78 (100)	
Tertiary	60 (56.6)	46 (43.4)	106 (100)	

Mother's occupation				
Unemployed	38 (67.9)	18 (32.1)	56 (100)	8.39 **(0.04)**
Public/civil servant	77 (57)	58 (43)	135 (100)	
Trader	154 (70)	66 (30)	220 (100)	
Retired	6 (46.2)	7 (53.8)	13 (100)	

Father's occupation				
Unemployed	18 (85.7)	3 (14.3)	21 (100)	10.88 **(0.01)**
Public/civil servant	117 (60)	78 (40)	195 (100)	
Farmer	129 (69.7)	56 (30.3)	185 (100)	
Retired	11 (47.8)	12 (52.2)	23 (100)	

**Table 8:** Association between the odds of good knowledge on the IFAS program and sociodemographic characteristics of respondents.

Dependent variable: good knowledge on the IFAS program							
Independent variables	Frequency (%)	Unadjusted			Adjusted		
		OR	95% CI	*p* value	OR	95% CI	*p* value
Mother's level of education							
No formal education	204 (48.1)	Reference					
Basic	100 (23.6)	0.96	0.57, 1.61	0.88	1.00	0.59, 1.70	1.00
Secondary	60 (14.2)	2.09	1.16, 3.77	**0.01**	2.19	1.81, 4.07	**0.01**
Tertiary	60 (14.2)	1.83	1.02, 3.30	**0.04**	1.25	0.64, 2.42	0.51

Mother's occupation							
Unemployed	56 (13.2)	0.63	0.33, 1.21	0.17	0.64	0.31, 1.31	0.22
Public/civil servant	135 (31.8)	Reference					
Trader	220 (51.9)	0.57	0.36, 0.89	**0.01**	0.66	0.39, 1.10	0.11
Retired	13 (3.1)	1.55	0.49, 4.85	0.45	1.25	0.37, 4.29	0.72

Father's occupation							
Unemployed	21 (5.0)	0.25	0.07, 0.88	**0.03**	0.29	0.08, 1.06	0.06
Public/civil servant	195 (46)	Reference					
Farmer	185 (43.6)	0.65	0.43, 1.00	0.05	0.81	0.51, 1.29	0.37
Retired	23 (5.4)	1.64	0.69, 3.89	0.27	1.79	0.70, 4.60	0.22

## Data Availability

The data used to support the findings of this study are available from the corresponding author upon request.
